# 4-Hydroxychalcone Inhibits Human Coronavirus HCoV-OC43 by Targeting EGFR/AKT/ERK1/2 Signaling Pathway

**DOI:** 10.3390/v17081028

**Published:** 2025-07-23

**Authors:** Yuanyuan Huang, Jieyu Li, Qiting Luo, Yuexiang Dai, Xinyi Luo, Jiapeng Xu, Wei Ye, Xinrui Zhou, Jiayi Diao, Zhe Ren, Ge Liu, Zhendan He, Zhiping Wang, Yifei Wang, Qinchang Zhu

**Affiliations:** 1 School of Pharmacy, Guangdong Pharmaceutical University, Guangzhou 510006, China; hyyaaa6@163.com (Y.H.); lijy2507@163.com (J.L.); 2112140258@stu.gdpu.edu.cn (Y.D.); 2 College of Pharmacy, Shenzhen Technology University, Shenzhen 518118, China; 2310415071@stumail.sztu.edu.cn (Q.L.); 18319399331@163.com (X.L.); xujiapeng2022@163.com (J.X.); yewei2000v@163.com (W.Y.); zhouxr1629@163.com (X.Z.); djy18018022302@163.com (J.D.); liuge@sztu.edu.cn (G.L.); hezhendan@sztu.edu.cn (Z.H.); 3 Guangdong Province Key Laboratory of Bioengineering Medicine, Key Laboratory of Innovative Technology Research on Natural Products and Cosmetics Raw Materials, College of Life Science and Technology, Jinan University, Guangzhou 510632, China; rz62@163.com

**Keywords:** human coronavirus, flavonoids, 4-hydroxychalcone, antiviral activity, EGFR, EGFR/AKT/ERK1/2 signaling pathway

## Abstract

Human coronaviruses are a group of viruses that continue to threaten human health. In this study, we investigated the antiviral activity of 4-hydroxychalcone (4HCH), a chalcone derivative, against human coronavirus HCoV-OC43. We found that 4HCH significantly inhibited the cytopathic effect, reduced viral protein and RNA levels in infected cells, and increased the survival rate of HCoV-OC43-infected suckling mice. Mechanistically, 4HCH targets the early stages of viral infection by binding to the epidermal growth factor receptor (EGFR) and inhibiting the EGFR/AKT/ERK1/2 signaling pathway, thereby suppressing viral replication. Additionally, 4HCH significantly reduced the production of pro-inflammatory cytokines and chemokines in both HCoV-OC43-infected RD cells and a suckling mouse model. Our findings demonstrate that 4HCH exhibits potent antiviral activity both in vitro and in vivo, suggesting its potential as a therapeutic agent against human coronaviruses. This study highlights EGFR as a promising host target for antiviral drug development and positions 4HCH as a candidate for further investigation in the treatment of coronavirus infections.

## 1. Introduction

Human coronaviruses (HCoVs) are a group of enveloped, positive-sense, single-stranded RNA viruses that infect humans and are known for their high mutation rates. Belonging to the Coronaviridae family, coronaviruses are classified into four genera: Alpha-, Beta-, Gamma-, and Deltacoronavirus [1]. To date, seven coronaviruses capable of infecting humans have been identified, with Beta-coronaviruses such as SARS-CoV, MERS-CoV, and SARS-CoV-2 posing the most significant threats to global health due to their severe complications and high mortality rates. The ongoing COVID-19 pandemic, caused by SARS-CoV-2, has underscored the urgent need for effective antiviral therapies to combat emerging and re-emerging coronaviruses [2,3,4].

Among the human coronaviruses, HCoV-OC43, a Beta-coronavirus first identified in the mid-1960s, is a major cause of respiratory infections [5]. While it typically induces mild upper respiratory symptoms such as cough, fever, and pharyngitis, HCoV-OC43 can also lead to severe lower respiratory tract infections, including bronchitis and pneumonia, particularly in infants, the elderly, and immunocompromised individuals. Notably, HCoV-OC43 exhibits neuroinvasive potential and has been associated with fatal cases of encephalitis [6]. Given the rapid mutation rate of coronaviruses, the efficacy of existing antiviral drugs and vaccines is frequently diminished, underscoring the urgent need to develop novel antiviral agents with distinct mechanisms of action [7,8,9]. HCoV-OC43 has emerged as a valuable model for studying coronavirus pathogenesis and screening antiviral compounds. Its use circumvents the stringent biosafety level 3 (BSL-3) requirements associated with SARS-CoV-2 research, enabling rapid identification of potential antiviral candidates [10].

Natural products, particularly flavonoids, have garnered significant attention due to their broad biological activities, including antiviral properties [11,12,13,14]. Chalcones, a subclass of flavonoids characterized by their α, β-unsaturated ketone structure, are particularly attractive due to their structural simplicity, ease of synthesis, and versatility in binding to various biological targets. These compounds exhibit a wide range of pharmacological activities, including anti-tumor, anti-inflammatory, antibacterial, and antiviral effects, making them promising candidates for drug development [15,16,17,18].

Recent studies have highlighted the potential of chalcone derivatives as antiviral agents. For instance, Chen et al. demonstrated that synthetic chalcone derivatives inhibit SARS-CoV-2 replication and exhibit anti-inflammatory effects [19]. Similarly, Herzog et al. reported that xanthohumol and 6-prenylnaringenin, both chalcone derivatives, effectively suppress SARS-CoV-2 replication in vitro [20]. Natural chalcones such as cardamonin and licochalcone B have also shown potent antiviral activity against HCoV-OC43 and SARS-CoV-2, respectively [21,22]. Through high-throughput screening, 4′-hydroxychalcone was identified as a broad-spectrum antiviral compound demonstrating efficacy against multiple coronaviruses, including HCoV-OC43, HCoV-NL63, MHV-A59, and MERS-CoV [23]. However, its therapeutic potential remains to be validated through in vivo studies, and the underlying molecular mechanisms require further investigation.

4-Hydroxychalcone (4HCH), an isomer of 4′-hydroxychalcone with a distinct chemical structure, is primarily derived from the roots of *Glycyrrhiza glabra* (*licorice*) and has been extensively studied for its diverse biological activities, including anti-angiogenic, antihypertensive, and anti-inflammatory effects [24,25]. Recent preclinical studies have demonstrated that 4HCH administration significantly attenuates Ang II-induced hypertension, mitigates cardiac remodeling, and improves cardiac function in murine models [26]. However, its antiviral potential, particularly against coronaviruses, remains underexplored.

In this study, we investigated the antiviral activity of 4HCH against HCoV-OC43 and elucidated its underlying molecular mechanisms. We demonstrate that 4HCH significantly inhibits viral replication in vitro and in vivo by targeting the epidermal growth factor receptor (EGFR) and modulating the EGFR/AKT/ERK1/2 signaling pathway. Additionally, 4HCH attenuates the production of pro-inflammatory cytokines and chemokines in both HCoV-OC43-infected cells and a suckling mouse model, suggesting its dual role as an antiviral and anti-inflammatory agent. Our findings highlight EGFR as a promising host target for antiviral drug development and position 4HCH as a potential therapeutic candidate for the treatment of coronavirus infections. This study not only provides new insights into the antiviral mechanisms of chalcone derivatives but also underscores the importance of host-targeting strategies in combating emerging and re-emerging viral pathogens.

## 2. Materials and Methods

### 2.1. Chemicals, Cells, and Viruses

4-Hydroxychalcone (4HCH) (CAS: 20426-12-4, purity > 99%) was purchased from TargetMol (Shanghai, China). Remdesivir (RDV) (CAS: 1809249-37-3) and Vandetanib (CAS: 443913-73-3) were obtained from MedChemExpress (MCE, Shanghai, China), with purities exceeding 99%. Human malignant embryonic rhabdomyoma (RD) cells (ATCC CCL-136, Manassas, VA, USA) were cultured in Dulbecco’s Modified Eagle Medium (DMEM, high glucose, Cytiva, Uppsala, Sweden) supplemented with 10% fetal bovine serum (FBS, Excell, Suzhou, China) and 1% penicillin-streptomycin (Gibco, New York, NY, USA) at 37 °C in a humidified atmosphere containing 5% CO_2_. Human coronavirus HCoV-OC43 (ATCC VR-1558, Manassas, VA, USA) was propagated in RD cells at 35 °C. The initial titer of the virus is 4.5 × 10^7^ PFU/mL. Viral titers were determined by plaque assay and expressed as plaque-forming units per milliliter (PFU/mL).

### 2.2. Cytotoxicity and Cytopathic Effect (CPE) Inhibition Assays

The cytotoxicity of 4HCH was assessed using the AlamarBlue assay. Briefly, RD cells were seeded at a density of 2 × 10^4^ cells/well in 96-well plates and incubated for 24 h. Cells were then treated with varying concentrations of 4HCH (6.25–100 µM) for 72 h. After treatment, 10% AlamarBlue solution (SAICHI, Beijing, China) was added to each well, and the plates were incubated at 37 °C for 2 h. Fluorescence was measured using a microplate reader (BioTek Epoch 2, Thermo Scientific, Walsham, MA, USA) at excitation/emission wavelengths of 530/590 nm. The 50% cytotoxic concentration (CC_50_) was calculated using nonlinear regression analysis.

To evaluate the antiviral activity of 4HCH, RD cells were infected with HCoV-OC43 at a multiplicity of infection (MOI) of 1 and treated with 4HCH at concentrations ranging from 1.56 to 50 µM simultaneously. After incubation of HCoV-OC43 with different concentrations of 4HCH at 35 °C for 72 h, the inhibition of CPE was evaluated by microscopy and quantified by AlamarBlue assay. The 50% inhibitory concentration (IC_50_) was determined as previously described [27].

### 2.3. Mouse Model of HCoV-OC43 Infection

All animal experiments were approved by the Animal Care and Use Committee of Rongwan Biological Laboratory Animal Center (Shenzhen, China) and conducted in accordance with institutional guidelines. Specific pathogen-free (SPF) C57BL/6 suckling mice (4–6 g, 10 days old, sex undetermined) were obtained from Guangdong Zhuhai Baishitong Biotechnology Co., Ltd. (Zhuhai, China), and were randomly divided into four groups (*n* = 12 per group): control (Ctrl), infection (HCoV-OC43), 4HCH treatment (HCoV-OC43 + 4HCH), and RDV treatment (HCoV-OC43 + 4HCH). Mice were intranasally infected with 10^6^ PFU of HCoV-OC43 and treated intraperitoneally with 4HCH (20 mg/kg) or RDV (20 mg/kg) once daily for 9 consecutive days. Control groups received placebo: 2% DMSO (Amresco, Darmstadt, Germany), 5% Tween-80 (Solarbio, Beijing, China), 40% PEG-300 (MCE, Shanghai, China), and 53% saline (Kelun, Chengdu, China). Survival rates and body weights were monitored daily. On day 4 post-infection, brain tissues were collected to assess viral load and viral N protein expression. This was accomplished using RT-qPCR and Western blot analysis, respectively.

### 2.4. Time of Addition and Plaque Reduction Assay

RD cells were co-incubated with HCoV-OC43 for 2 h, then the virus solution was removed, and the marker was removed for 0 h. After washing three holes with PBS, a fixed concentration of 10 μM 4HCH was added to continue culture. Three holes were washed and drugs were added at 1, 2, 4, 8, 10, 12 and 14 h, as different steps and time points in the life cycle of virus infection. Cell samples were collected at 14 h after labeling, and the sample solution at different time stages was subjected to plaque titration, and the spots were calculated.

RD cells were seeded in 24-well plates at 4 × 10^5^ cells/well and incubated for 24 h to form monolayers. Pretreatment: 4HCH was pre-incubated with virus for 1 h, and then co-incubated with cells at 37 °C for 2 h. Co-treatment: 4HCH and virus were added to the cells and incubated at 37 °C for 2 h. Subsequently, the above inoculums were replaced with a maintenance medium containing 1.2% methylcellulose (Sigma-Aldrich, Darmstadt, Germany). Post-treatment: The virus was incubated with the cells at 37 °C for 2 h, and the inoculum was replaced with a maintenance medium containing 1.2% methylcellulose (Sigma-Aldrich, Darmstadt, Germany) and 4HCH was added. Subsequently, the cells were incubated at 35 °C for 96 h, fixed with 10% formaldehyde (Solarbio, Beijing, China), and stained with 0.1% crystal violet (Biyuntian, Shanghai, China). Plaques were counted and the percentage of plaque reduction was calculated.

### 2.5. RNA Isolation and Quantitative Reverse Transcription PCR (RT-qPCR)

Total RNA was extracted from infected or uninfected RD cells or brain tissues of suckling mice infected with HCoV-OC43 and treated with 4HCH using the Takara MiniBEST Universal RNA Extraction Kit (Takara Bio, Otsu, Japan). cDNA was synthesized using the PrimeScript RT Reagent Kit (Takara Bio, Otsu, Japan). RT-qPCR was performed on a QuantStudio 5 Real-Time PCR System (Applied Biosystems, Thermo Scientific, Walsham, MA, USA) using SYBR Green Master Mix (Takara Bio, Otsu, Japan). The mRNA levels of the HCoV-OC43 nucleocapsid (N) gene and pro-inflammatory cytokines/chemokines (TNFα, IL-6, CXCL-2, CXCL-3, CXCL-8/IL-8, and CXCL-10) were quantified. β-Actin served as the internal control. Primer sequences for quantification in RD cells are listed in Table 1.

### 2.6. Western Blot Analysis

Cells were lysed in RIPA buffer, and protein concentrations were determined using the Bradford assay. Equal amounts of protein were denatured in 5× loading buffer at 95 °C for 5 min, separated by SDS-PAGE, and transferred to PVDF membranes. Membranes were probed with primary antibodies against phospho-EGFR (CST, 3777, Denver, CO, USA), EGFR (CST, 54359), phospho-AKT (CST, 4060), AKT (CST, 4691), phospho-ERK1/2 (CST, 4370), ERK1/2 (CST, 4695), GAPDH (CST, 2118), and HCoV-OC43 nucleocapsid protein (Sino Biological, 40643-T62, Beijing, China). Protein bands were visualized using enhanced chemiluminescence (ECL, Epizyme, Shanghai, China) and quantified using ImageJ 1.54g software.

### 2.7. Transcriptome and Connectivity Map (cMap) Analysis

Total RNA was extracted from RD cells treated with 10 µM 4HCH or DMSO for 24 h using TRIzol reagent (GBCBIO, Guangzhou, China). RNA-seq libraries were prepared and sequenced on an Illumina platform by Genedenovo Biotechnology Co., Ltd. (Guangzhou, China). Differentially expressed genes (DEGs) were identified using DESeq2 with adjusted *p* < 0.05 and fold change ≥ 2. Functional enrichment analysis was performed using the Connectivity Map (cMAP) database to identify potential molecular targets and pathways as previously described [28].

### 2.8. Cellular Thermal Shift Assay (CETSA)

RD cells were lysed in M-PER buffer (Thermo Scientific, Walsham, MA, USA) containing 1% PMSF (Solarbio, Beijing, China) after 8 h infection with HCoV-OC43. Lysates were treated with 4HCH or DMSO for 2 h at room temperature, divided into aliquots, and heated at temperatures ranging from 38 °C to 58 °C for 3 min. Samples were cooled, centrifuged, and analyzed by Western blot to assess EGFR thermal stability.

### 2.9. Molecular Docking

The crystal structure of EGFR (PDB ID: 5D41) was obtained from the Protein Data Bank. Protein preparation and molecular docking simulations were performed using Schrödinger Maestro (2018). Potential binding sites were identified using the SiteMap module, and binding free energy were calculated to evaluate the binding affinity of 4HCH or Vandetanib to EGFR.

### 2.10. Statistical Analysis

All data are presented as the mean ± standard deviation (SD). All statistical analyses were conducted using GraphPad Prism 8.0.2 software (GraphPad Software, San Diego, CA, USA). The CC_50_ and IC_50_ values were calculated using nonlinear regression analysis (four-parameter logistic model) in the software. Statistical significance was denoted as * *p* < 0.05, ** *p* < 0.01, *** *p* < 0.001, **** *p* < 0.0001, or ns (not significant). The statistical method, either one-way ANOVA or Student’s *t*-test, was selected based on the experimental design and is specified in the figure legends. Survival curves were generated using the Kaplan–Meier method and evaluated for statistical significance using the log-rank (Mantel–Cox) test.

## 3. Results

### 3.1. 4HCH Exhibits Potent Antiviral Activity Against HCoV-OC43 In Vitro

**Figure 1:**
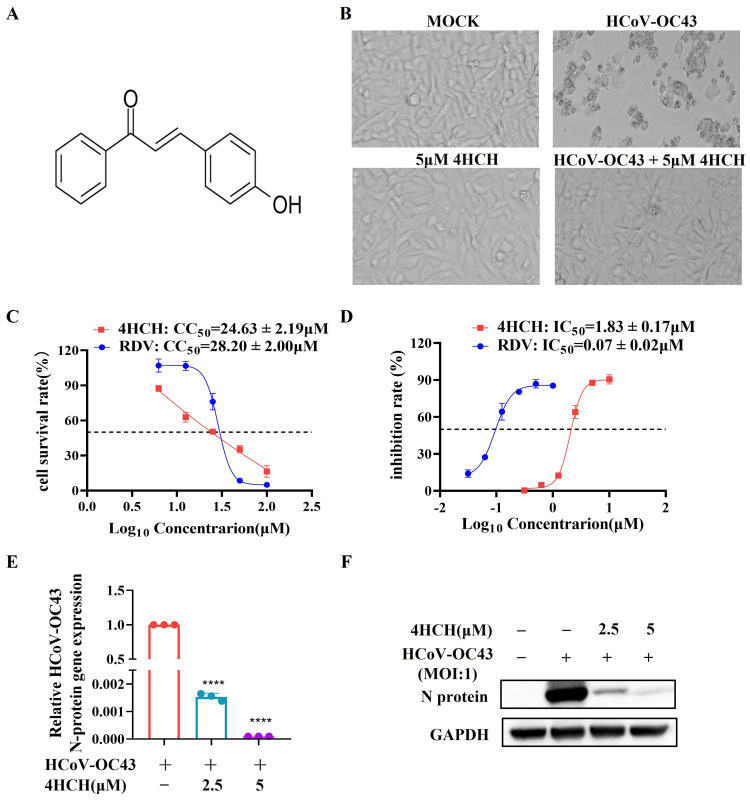
4HCH inhibits HCoV-OC43 replication in vitro. (**A**) Chemical structure of 4-hydroxychalcone (4HCH). (**B**) Representative images of RD cells at 3 days post-infection (dpi) in control group, 5 µM 4HCH-treated group, HCoV-OC43-infected group, and HCoV-OC43-infected group treated with 5 µM 4HCH. (**C**) Cytotoxicity (CC_50_) of 4HCH and remdesivir (RDV) against HCoV-OC43 in RD cells, determined using AlamarBlue assay. (**D**) Antiviral activity (IC_50_) of RDV and 4HCH against HCoV-OC43 in RD cells, determined using AlamarBlue assay. (**E**,**F**) Viral nucleocapsid (N) mRNA and protein levels in HCoV-OC43-infected RD cells treated with 4HCH for 48 h, assessed by RT-qPCR and Western blot, respectively. β-Actin and GAPDH served as internal controls. Data are presented as mean ± SD. All experiments were repeated independently three times. Student’s *t*-test was used to assess statistical significance of differences between untreated and 4HCH-treated groups. **** *p* < 0.0001 vs. HCoV-OC43-infected group.

To evaluate the antiviral activity of 4-hydroxychalcone (4HCH) (Figure 1A), RD cells were infected with HCoV-OC43 and treated with varying concentrations of 4HCH. Microscopic examination confirmed the inhibition of CPE in cells treated with 5 µM 4HCH (Figure 1B). Its 50% cytotoxic concentration (CC_50_) in RD cells, as determined by the AlamarBlue assay, is 24.63 ± 2.19 µM. This CC_50_ value is comparable to that of remdesivir (RDV), a known antiviral and positive control, which has a CC_50_ of 28.20 ± 2.00 µM (Figure 1C). More importantly, 4HCH significantly inhibited HCoV-OC43-induced CPE in a dose-dependent manner, with an IC_50_ value of 1.83 ± 0.17 µM. While RDV exhibited a lower IC_50_ of 0.07 ± 0.02 µM, the effectiveness of 4HCH is clearly demonstrated (Figure 1D). Further analyses using RT-qPCR (Figure 1E) and Western blot (Figure 1F) confirmed that 4HCH treatment significantly reduced both the mRNA levels of the viral nucleocapsid (N) gene and the expression of the N protein in infected cells. These findings collectively indicate that 4HCH effectively suppresses HCoV-OC43 replication in vitro.

### 3.2. 4HCH Demonstrates Antiviral Efficacy in a Suckling Mouse Model

**Figure 2:**
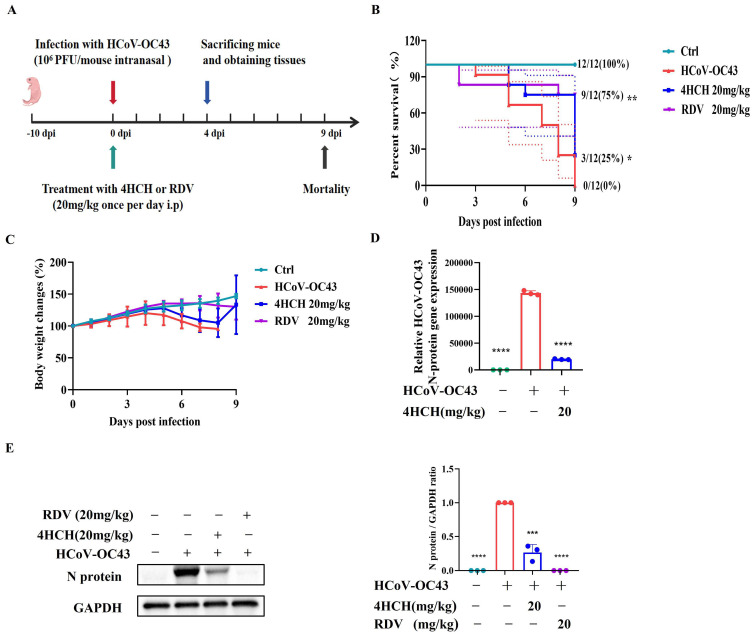
4HCH exhibits antiviral activity in a suckling mouse model of HCoV-OC43 infection. (**A**) Experimental design. Ten-day-old C57BL/6 suckling mice were intranasally infected with 10^6^ PFU of HCoV-OC43 and treated daily with 4HCH (20 mg/kg), RDV (20 mg/kg), or placebo for 9 days. (**B**) Survival rates of mice monitored daily until 9 days post-infection (dpi) (*n* = 12). (**C**) Changes in body weight of infected mice (*n* = 12). (**D**) Viral nucleocapsid (N) gene levels in brain tissues of infected mice collected at 4 dpi, assessed by RT-qPCR. GAPDH served as an internal control (*n* = 3). (**E**) Viral N protein levels in brain tissues of infected mice collected at 4 dpi, assessed by Western blot (*n* = 3). GAPDH served as an internal control. Data are presented as mean ± SD. Survival curves were generated using the Kaplan–Meier method and analyzed for statistical significance using the log-rank test. * *p* < 0.05, ** *p* < 0.01, *** *p* < 0.001, **** *p* < 0.0001 vs. HCoV-OC43-infected group.

To assess the in vivo antiviral activity of 4HCH, 10-day-old C57BL/6 suckling mice were intranasally infected with 10^6^ PFU of HCoV-OC43 and treated daily with 4HCH (20 mg/kg) or RDV (20 mg/kg) for 9 days (Figure 2A). In the untreated infection group, mortality began on day 3 post-infection, with all mice succumbing to the infection by day 9. In contrast, both 4HCH and RDV treatments significantly improved survival rates. By day 8 post-infection, the survival rate in the 4HCH group was 75%, compared to 25% in the infection-only group, and 80% in the RDV group (Figure 2B). Although 4HCH treatment significantly improved survival, it did not restore body weight to baseline levels (Figure 2C).

To further validate the antiviral effect, brain tissues were collected on day 4 post-infection, and viral N gene and protein levels were quantified by RT-qPCR and Western blot, respectively. 4HCH treatments significantly reduced viral load and viral protein expression compared to the untreated group (Figure 2D,E). These findings confirm the in vivo efficacy of 4HCH against HCoV-OC43.

### 3.3. 4HCH Targets the Post-Entry Phase of HCoV-OC43 Infection

**Figure 3:**
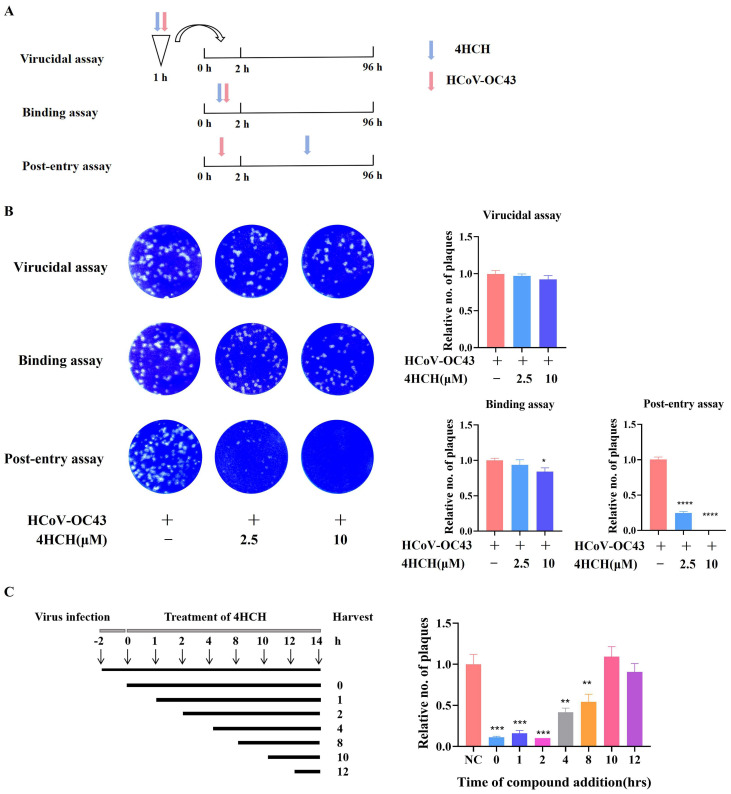
4HCH targets the post-entry phase of HCoV-OC43 infection. (**A**,**B**) Experimental setup to determine if 4HCH directly inactivates virus particles or blocks viral attachment. RD cells were treated with 4HCH under three conditions: pre-treatment (4HCH + virus pre-incubation for 1 h), co-treatment (4HCH + virus co-incubation for 2 h), and post-treatment (4HCH added after viral infection). Plaque formation was assessed after 4 days. (**C**) Time-of-addition analysis. RD cells were infected with HCoV-OC43, and 4HCH was added at 0, 1, 2, 4, 8, 10, and 12 h post-infection. Viral titers in the supernatant were determined by plaque assay. Data are presented as mean ± SD. A Student’s *t*-test was used to assess the statistical significance of differences between untreated and 4HCH-treated groups. * *p* < 0.05, ** *p* < 0.01, *** *p* < 0.001, **** *p* < 0.0001 vs. HCoV-OC43-infected group. All experiments were repeated three times independently.

To elucidate the stage of the viral life cycle targeted by 4HCH, we performed time-of-addition experiments (Figure 3A). Pre-treatment (4HCH + virus pre-incubation for 1 h) and co-treatment (4HCH + virus co-incubation for 2 h) did not significantly reduce plaque formation, indicating that 4HCH does not directly inactivate viral particles or block viral attachment (Figure 3B). However, post-treatment (4HCH added after viral infection) significantly inhibited plaque formation, with 10 µM 4HCH completely eliminating viral plaques (Figure 3B). Further analysis revealed that 4HCH exerted its antiviral effect when added within 0–8 h post-infection (Figure 3C), suggesting that it targets the early stages of viral replication following viral entry.

### 3.4. 4HCH Inhibits the EGFR/AKT/ERK1/2 Signaling Pathway

**Figure 4:**
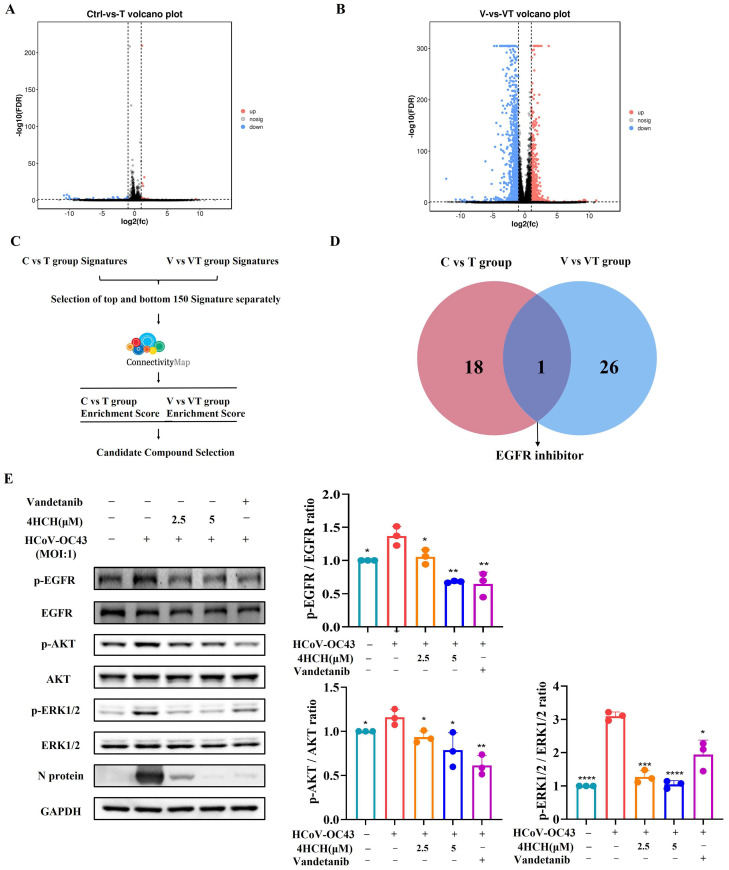
4HCH inhibits the EGFR/AKT/ERK1/2 signaling pathway. (**A**,**B**) Volcano plots showing differentially expressed genes (DEGs) in RD cells treated with 4HCH under normal (C vs. T) and HCoV-OC43-infected (V vs. VT) conditions. DEGs were selected based on adjusted *p* < 0.05 and fold change > 2. (**C**) Schematic of the Connectivity Map (cMAP) analysis to identify compounds with mechanisms of action similar to 4HCH. (**D**) Functional overlap analysis of cMAP-identified compounds, highlighting EGFR inhibitors. (**E**) Western blot analysis of EGFR, phosphorylated EGFR (p-EGFR), AKT, phosphorylated AKT (p-AKT), ERK1/2, and phosphorylated ERK1/2 (p-ERK1/2) in HCoV-OC43-infected RD cells treated with 4HCH or the EGFR inhibitor Vandetanib for 24 h. GAPDH served as an internal control. Data are presented as mean ± standard deviation (SD). Statistical significance is indicated as * *p* < 0.05, ** *p* < 0.01, *** *p* < 0.001, and **** *p* < 0.0001 compared to the untreated HCoV-OC43-infected group. All experiments were independently performed in triplicate. A Student’s *t*-test was used to evaluate statistical significance between the untreated HCoV-OC43-infected group and the 4HCH- or Vandetanib-treated groups.

To further explore the antiviral mechanism of 4HCH, RD cells, or RD cells infected with the virus, were treated with 4HCH or DMSO for 24 h. The cells were then harvested, and next-generation sequencing (NGS) was performed to identify differentially expressed genes (DEGs). In the normal cell dosing group (C vs. T), a total of 48 DEGs were identified, including 18 up-regulated genes and 30 down-regulated genes (Figure 4A). In the viral infection dosing group (V vs. VT), 2422 DEGs were identified, with 935 up-regulated genes and 1487 down-regulated genes (Figure 4B).

To further analyze the potential antiviral mechanism of 4HCH, we used the Connectivity Map (cMAP) database, which links small molecules, genes, cells, and diseases. Compounds that show similar expression patterns may share similar pharmacological mechanisms. We input the top 150 significantly up-regulated and down-regulated genes from both the normal and viral infection dosing groups into the cMAP database for analysis (Figure 4C). A connectivity score closer to +1 indicates a stronger similarity in the mechanism of action, while a score closer to −1 indicates the opposite. Based on the top 50 similar compounds from both groups, our analysis suggests that the mechanism of 4HCH might resemble that of EGFR inhibitors (Figure 4D). It is important to note that many respiratory viruses, including SARS-CoV-2, can induce EGFR activation in airway epithelial cells [29,30,31]. In one study, EGFR inhibitors were found to inhibit the early-stage spread of SARS-CoV-2, suggesting their potential as promising antiviral candidates [29]. EGFR inhibitors have been shown to reduce the phosphorylation of EGFR, ERK, and AKT [32,33]. In our study, we observed that 4HCH treatment, along with the EGFR inhibitor Vandetanib, significantly reduced the phosphorylation levels of EGFR, AKT, and ERK1/2 in RD cells infected with HCoV-OC43 (Figure 4E). The total AKT expression itself did not significantly change across conditions (*p* > 0.05), indicating the experimental treatments did not alter its abundance. Our conclusions regarding AKT signaling activity are based primarily on the phosphorylation status of AKT (*p*-AKT). While the absolute p-AKT/AKT ratio change in Figure 4 may appear modest, we observed statistically significant differences (*p* < 0.05) in p-AKT levels relative to the relevant control groups under specific experimental conditions. This altered phosphorylation state is a key indicator of pathway modulation. Additionally, Vandetanib treatment also significantly inhibited the expression of viral proteins (Figure 4E). These results suggest that 4HCH inhibits HCoV-OC43 replication by targeting the EGFR/AKT/ERK1/2 signaling pathway.

### 3.5. 4HCH Likely Interacts with EGFR

**Figure 5:**
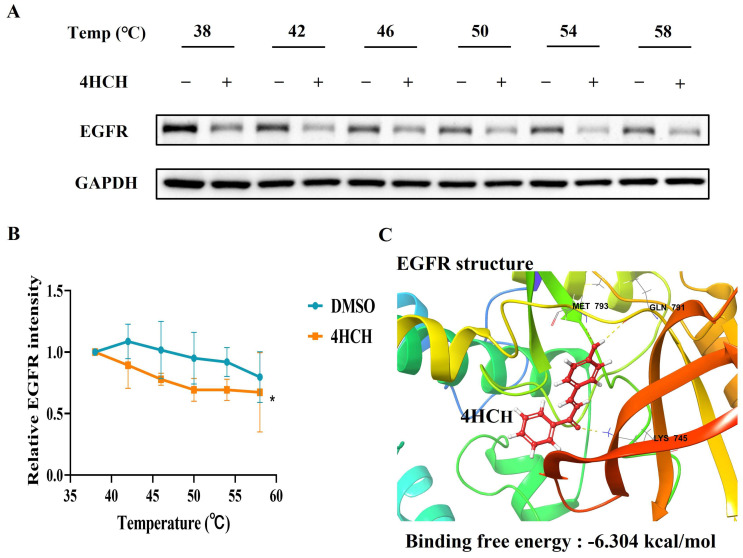
4HCH likely interacts with EGFR. (**A**,**B**) Cellular thermal shift assay (CETSA) showing the thermal stabilization of EGFR in HCoV-OC43-infected RD cells treated with 4HCH. GAPDH served as a control. (**C**) Molecular docking analysis of 4HCH binding to EGFR, with key residues MET793, GLN791, and LYS745 forming hydrogen bonds. The binding energy of 4HCH to EGFR is −6.304 kcal/mol. Data are presented as mean ± standard deviation (SD). Statistical significance is indicated as * *p* < 0.05 compared to the DMSO control group. A Student’s *t*-test was used to evaluate statistical significance between the DMSO control group and the 4HCH-treated group.

To validate EGFR as a direct target of 4HCH, we performed a cellular thermal shift assay (CETSA) as previously described [34,35]. Treatment with 4HCH reduced the thermal stability of EGFR in HCoV-OC43-infected RD cells, indicating direct binding (Figure 5A,B). Molecular docking simulations further supported this interaction, revealing that 4HCH forms hydrogen bonds with key residues (MET 793, GLN 791, and LYS 745) in the EGFR binding pocket (Figure 5C). The binding free energy of 4HCH to EGFR was calculated to be −6.304 kcal/mol, comparable to that of EGFR inhibitor Vandetanib (−5.868 kcal/mol). These results collectively demonstrate that 4HCH likely targets EGFR, contributing to its antiviral efficacy against HCoV-OC43.

### 3.6. 4HCH Suppresses Cytokine and Chemokine Expression Induced by HCoV-OC43 Infection

**Figure 6:**
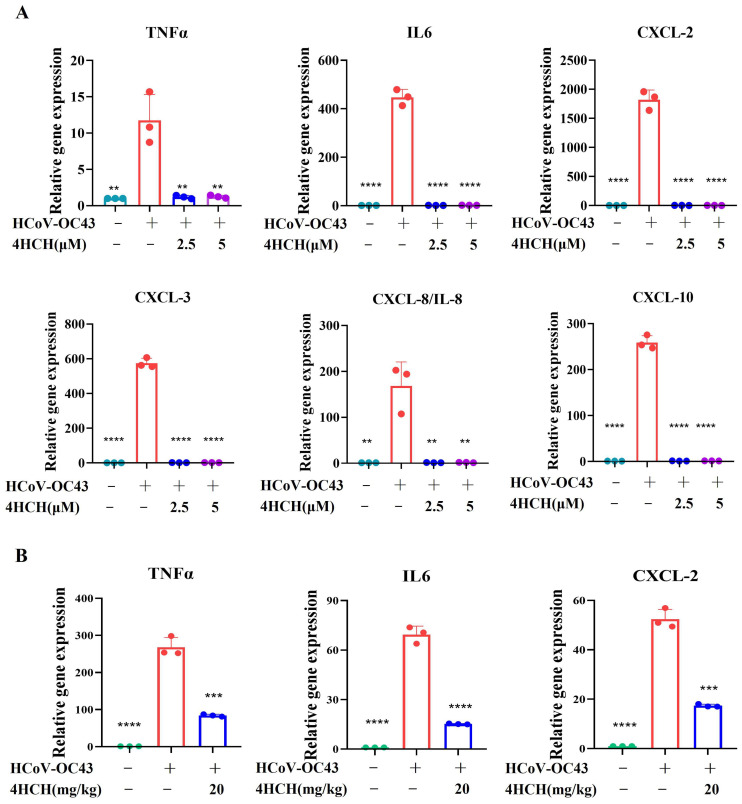
4HCH suppresses pro-inflammatory cytokine and chemokine expression in HCoV-OC43-infected cells. (**A**) RT-qPCR analysis of mRNA levels of TNFα, IL-6, CXCL-2, CXCL-3, CXCL-8/IL-8, and CXCL-10 in RD cells infected with HCoV-OC43 and treated with 4HCH (2.5 µM and 5 µM). (**B**) RT-qPCR analysis of mRNA levels of TNFα, IL-6, and CXCL-2 in the brain tissues of suckling mice infected with HCoV-OC43 and treated with 4HCH (20 mg/kg). Data are presented as mean ± SD. A Student’s *t*-test was used to assess the statistical significance of differences between untreated and 4HCH-treated groups. ** *p* < 0.01, *** *p* < 0.001, **** *p* < 0.0001 vs. HCoV-OC43-infected group. All experiments were repeated three times independently.

Coronavirus infections often trigger a strong inflammatory response, characterized by the elevated production of cytokines and chemokines [36,37]. To investigate whether 4HCH could inhibit the expression of pro-inflammatory mediators in HCoV-OC43-infected cells, we measured the mRNA levels of several key cytokines and chemokines in vitro and in vivo. As shown in Figure 6A, the viral infection group exhibited significantly increased mRNA levels of IL-6, TNFα, CXCL-2, CXCL-3, CXCL-8/IL-8, and CXCL-10 compared to the NC group. In contrast, treatment with 4HCH (at 2.5 μM and 5 μM) significantly reduced the expression of these cytokines and chemokines in the virus-infected cells (*p* < 0.001). In addition to the in vitro evaluation, we investigated the in vivo effects of 4HCH on inflammatory factor expression. Specifically, we analyzed cytokine and chemokine gene expression in brain tissues of HCoV-OC43-infected suckling mice treated with 4HCH (20 mg/kg). The results demonstrated that 4HCH significantly reduced the expression of TNFα, IL-6, and CXCL2 (Figure 6B), consistent with observations from in vitro infected cells. These results suggest that 4HCH may act as an effective anti-inflammatory agent.

## 4. Discussion

Respiratory diseases caused by coronavirus infections, including the ongoing COVID-19 pandemic, remain a major global health challenge. HCoV-OC43, a Beta-coronavirus, is a major cause of respiratory infections, causing symptoms ranging from mild upper respiratory illness to severe lower respiratory tract infections such as bronchitis and pneumonia. These severe manifestations occur particularly in infants, the elderly, and immunocompromised individuals. However, no specific antiviral drugs are currently available against HCoV-OC43, highlighting an urgent need for the development of novel antiviral agents with distinct mechanisms of action. Furthermore, as coronavirus infections often trigger severe inflammatory responses, including cytokine storms, it is equally important to identify compounds that also possess anti-inflammatory properties, providing a dual therapeutic effect [7,8].

In this study, we provide compelling evidence that 4HCH, a chalcone derivative, exhibits potent antiviral activity against human coronavirus HCoV-OC43, both in vitro and in vivo. 4HCH acts by targeting the epidermal growth factor receptor (EGFR) and inhibiting the downstream AKT and ERK1/2 signaling pathways, which are crucial for viral replication. Viruses frequently co-opt the AKT and ERK1/2 signaling pathways to establish a cellular environment conducive to their replication. Activation of these pathways promotes critical processes including: host cell survival (delaying apoptosis), reprogramming of host cell metabolism (to provide energy and biosynthetic precursors), facilitation of viral genome replication and protein synthesis, and suppression of intrinsic host antiviral defenses. This host-targeting strategy contrasts with the mechanism of action of many direct-acting antivirals, which typically target viral enzymes or entry mechanisms. By modulating host cell signaling pathways, 4HCH presents a promising strategy to combat viral replication, offering potential broad-spectrum antiviral activity.

Previous studies have demonstrated that EGFR activation plays a key role in the infection of many respiratory viruses, including SARS-CoV-2 [29,31,38]. For example, Shin et al. showed that SARS-CoV-2 activates EGFR-mediated survival signals in airway epithelial cells, contributing to mitochondrial dysfunction and promoting viral replication [29]. In line with these findings, we observed that 4HCH treatment, along with the FDA-approved EGFR inhibitor Vandetanib, effectively reduced the activation of EGFR and its downstream pathways, leading to a significant inhibition of HCoV-OC43 replication. Notably, Vandetanib has demonstrated effective anti-SARS-CoV-2 activity both in vitro and in vivo [29]. Vandetanib, an approved EGFR inhibitor for medullary thyroid cancer, targets EGFR, VEGFR, and RET, offering broad efficacy, but is limited by adverse effects like diarrhea, rash, nausea, hypertension, and QT prolongation. In our in vitro cytotoxicity evaluation, vandetanib exhibited a CC_50_ value of 9.33 µM, indicating a toxicity level approximately 2–3 times higher than that of 4HCH, which showed a CC_50_ value of 24.6 µM. Preliminary data indicate 4HCH maintains potent EGFR inhibition, possibly matching vandetanib’s efficacy with fewer side effects. However, vandetanib’s established clinical profile contrasts with 4HCH’s early-stage development, where its kinase specificity and long-term safety need further study. While vandetanib’s multi-kinase inhibition suits complex cancers, 4HCH’s specificity may reduce off-target effects but limit its indications. Thus, 4HCH’s lower cytotoxicity makes it a promising candidate for further research to confirm its efficacy and safety.

In the CETSA experiment, treatment with 4HCH reduced the thermal stability of EGFR protein in RD cells infected with HCoV-OC43. Additionally, the binding energy of 4HCH to EGFR, determined through molecular docking simulations, is comparable to that of Vandetanib, suggesting that 4HCH targets EGFR in a similar manner to established EGFR inhibitors. Compared to direct-acting antivirals (DAAs), host-targeting antivirals (HTAs) offer advantages such as broader spectrum activity and a higher barrier to drug resistance.

Cytokine storms play a critical role in the development of fatal pneumonia and are a leading cause of mortality in coronavirus infections [39]. Acute respiratory distress syndrome (ARDS), a common immunopathological consequence of infections with SARS-CoV-2, SARS-CoV, and MERS-CoV, is often driven by cytokine storms. These storms involve an uncontrolled systemic inflammatory response characterized by the excessive release of pro-inflammatory cytokines (such as TNFα, IL-6, IL-1β, IL-12, IL-18, IL-33, TGF-β) and chemokines (including CCL-2, CCL-3, CCL-5, CXCL-8, and CXCL-10) by immune effector cells [40,41]. The resulting immune overreaction can cause severe tissue damage, leading to ARDS, multiple organ failure, and ultimately death in severe cases of SARS-CoV-2 infection. In our study, we found that 4HCH significantly inhibited the production of TNFα, IL-6, CXCL-2, CXCL-3, CXCL-8/IL-8, and CXCL-10 in RD cells infected with HCoV-OC43. To assess its in vivo effects on inflammatory mediator expression, we analyzed cytokine and chemokine gene levels in the brain tissues of HCoV-OC43-infected suckling mice treated with 4HCH. Notably, 4HCH treatment also significantly attenuated the expression of TNFα, IL-6, and CXCL-2 in vivo. While 4HCH suppressed cytokine and chemokine expression both in vitro and in vivo, the protective effects of 4HCH in infected animals cannot be attributed to immune suppression, given the immature immune responses in suckling mice. Instead, these effects are likely primarily due to its inhibition of viral replication. HCoV-OC43, a common human coronavirus, typically causes mild respiratory infections but can induce cytokine and chemokine responses in infected individuals, such as IL6, TNF-α, IL-8, CCL2 (MCP-1), and CCL5. Consistent with clinical observations, in vitro infection of RD cells and in vivo infection of suckling mice in our study similarly demonstrated significant upregulation of IL-6, TNF-α, CXCL2, CXCL3, CXCL8 (IL-8), and CXCL10. Nevertheless, its capacity to trigger severe cytokine storm syndromes—characterized by uncontrolled systemic inflammation—remains substantially lower than that of highly pathogenic coronaviruses such as SARS-CoV-2 or SARS-CoV.

4HCH’s dual mechanism of action, targeting both viral replication and inflammation, provides a strong rationale for its further development as a therapeutic candidate for coronavirus infections. Viral infections frequently cause pathology through excessive inflammatory responses. Given 4HCH’s notable anti-inflammatory properties, it holds promise as a complementary agent in combination therapies with other antiviral drugs, potentially enhancing overall therapeutic efficacy by addressing both viral replication and inflammation. Moreover, as it targets host cell signaling pathways rather than viral proteins, 4HCH may have a higher barrier to resistance, which is a key advantage over traditional DAAs. The ability to modulate host factors essential for viral replication also suggests that 4HCH could be effective against a range of coronaviruses, making it a promising candidate for broad-spectrum antiviral therapy.

Although 4HCH exhibits a relatively modest selectivity index (SI) of ~14 against HCoV-OC43, our study provides critical insights into its mechanism. Notably, while no approved antivirals target EGFR for coronavirus treatment, we identify EGFR as a potential target of 4HCH. This finding supports novel antiviral strategies leveraging EGFR inhibition. Importantly, 4HCH demonstrated significant efficacy against HCoV-OC43 both in vitro and in vivo. Given the unmet therapeutic need for coronaviruses, 4HCH represents a promising lead compound. To enable further optimization, we performed structure-activity relationship (SAR) analysis of chalcone derivatives (Supplementary Table S1). Structural modification of 4HCH thus holds promise for developing compounds with enhanced anti-HCoV-OC43 activity, although further investigation is required.

## 5. Conclusions

In summary, our study demonstrates that 4HCH exhibits potent antiviral activity against HCoV-OC43 by targeting the EGFR/AKT/ERK1/2 signaling pathway. The compound not only inhibits viral replication but also attenuates the production of pro-inflammatory cytokines. These findings underscore the importance of host-targeting strategies in the development of broad-spectrum antiviral therapies. Further investigation into the therapeutic potential of 4HCH and its derivatives is warranted to address the ongoing challenges posed by coronavirus infections.

## Figures and Tables

**Table 1:** Primer sequences.

Gene	Forward (5′-3′ Sequence)	Reverse (5′-3′ Sequence)
HCoV-OC43 N	AGCAACCAGGCTGATGTCAATACC	AGCAGACCTTCCTGAGCCTTCAAT
TNFα	GAACCCCGAGTGACAAGCCT	TATCTCTCAGCTCCACGCCAT
IL6	GCAATAACCACCCCTGACCCAA	GCTACATTTGCCGAAGAGCC
CXCL-2	CCGAAGTCATAGCCACACTCA	TGGATTTGCCATTTTTCAGCATCT
CXCL-3	TGAATGTAAGGTCCCCCGGA	CACCCTGCAGGAAGTGTCAA
CXCL-8	CAGTTTTGCCAAGGAGTGCT	ACTTCTCCACAACCCTCTGC
CXCL-10	TGAATCCAGAATCGAAGGCCA	TGCATCGATTTTGCTCCCCT
β-Actin	CTCACCATGGATGATGATATCGC	ATAGGAATCCTTCTGACCCATGC
TNFα (Mouse)	AGGGTCTGGGCCATAGAACT	CCACCACGCTCTTCTGTCTAC
IL6 (Mouse)	ATCCAGTTGCCTTCTTGGGACTGA	TAAGCCTCCGACTTGTGAAGTGGT
CXCL-2 (Mouse)	CACTCTCAAGGGCGGTCAA	AGGCACATCAGGTACGATCCA
GAPDH (Mouse)	CCTCGTCCCGTAGACAAAATG	TGAGGTCAATGAAGGGGTCGT

## Data Availability

The data supporting the findings of this study are available within the article and its Supplementary Materials.

## Supplementary Materials

The following supporting information can be downloaded at https://www.mdpi.com/article/10.3390/v17081028/s1, Table S1: SAR studies of chalcone and its derivatives against human coronavirus HCoV-OC43.

## Abbreviations

The following abbreviations are used in this manuscript:

4-HCH	4-hydroxychalcone
AKT	protein kinase B
ARDS	acute respiratory distress syndrome
CC50	50% cytotoxic concentration
CETSA	cellular thermal shift assay
cMAP	connectivity map
CPE	cytopathic effect
COVID-19	coronavirus disease 2019
EGFR	epidermal growth factor receptor
ERK1/2	extracellular regulated protein kinases 1/2
HCoV-OC43	human coronavirus OC43
HCoVs	human coronaviruses
IC50	50% inhibitory concentration
MAPK	mitogen-activated protein kinase
MERS-CoV	Middle East Respiratory Syndrome coronavirus
MHV-A59	mouse hepatitis virus A-59
MOI	multiplicity of infection
PFU	plaque forming units
RD	rhabdomyosarcoma
RDV	Remdesivir
RT-qPCR	quantitative real-time PCR
SARS-CoV	severe acute respiratory syndrome coronavirus
SARS-CoV-2	severe acute respiratory syndrome coronavirus 2

## References

[1] Cui J., Li F., Shi Z.-L. (2019). Origin and evolution of pathogenic coronaviruses. Nat. Rev. Microbiol..

[2] Tang G., Liu Z., Chen D. (2022). Human coronaviruses: Origin, host and receptor. J. Clin. Virol..

[3] Fung T.S., Liu D.X. (2019). Human Coronavirus: Host-Pathogen Interaction. Annu. Rev. Microbiol..

[4] Wang C., Horby P.W., Hayden F.G., Gao G.F. (2020). A novel coronavirus outbreak of global health concern. Lancet.

[5] Gaunt E.R., Hardie A., Claas E.C.J., Simmonds P., Templeton K.E. (2010). Epidemiology and clinical presentations of the four human coronaviruses 229E, HKU1, NL63, and OC43 detected over 3 years using a novel multi plex real-time PCR method. J. Clin. Microbiol..

[6] Nilsson A., Edner N., Albert J., Ternhag A. (2020). Fatal encephalitis associated with coronavirus OC43 in an immunocompromised child. Infect. Dis..

[7] Parums D.V. (2023). Editorial: The XBB.1.5 (‘Kraken’) Subvariant of Omicron SARS-CoV-2 and its Rapid Global Spread. Med. Sci. Monit..

[8] Wang Y., Long Y., Wang F., Li C., Liu W. (2023). Characterization of SARS-CoV-2 recombinants and emerging Omicron sublineages. Int. J. Med. Sci..

[9] Ao D., He X., Liu J., Xu L. (2020). Strategies for the development and approval of COVID-19 vaccines and therapeutics in the post-pandemic period. Signal Transduct. Target. Ther..

[10] Kim M.I., Lee C. (2023). Human Coronavirus OC43 as a Low-Risk Model to Study COVID-19. Viruses.

[11] Sopjani M., Falco F., Impellitteri F., Guarrasi V., Nguyen Thi X., Dërmaku-Sopjani M., Faggio C. (2004). Flavonoids derived from medicinal plants as a COVID-19 treatment. Phytother. Res..

[12] Yang J.-Y., Ma Y.-X., Liu Y., Peng X.-J., Chen X.-Z. (2024). A Comprehensive Review of Natural Flavonoids with Anti-SARS-CoV-2 Activity. Molecules.

[13] Pérez-Vargas J., Shapira T., Olmstead A.D., Villanueva I., Thompson C.A.H., Ennis S., Gao G., De Guzman J., Williams D.E., Wang M. (2023). Discovery of lead natural products for developing pan-SARS-CoV-2 therapeutics. Antivir. Res..

[14] Khalifa S.A.M., Yosri N., El-Mallah M.F., Ghonaim R., Guo Z., Musharraf S.G., Du M., Khatib A., Xiao J., Saeed A. (2021). Screening for natural and derived bio-active compounds in preclinical and clinical studies: One of the frontlines of fighting the coronaviruses pandemic. Phytomedicine.

[15] Zhuang C., Zhang W., Sheng C., Zhang W., Xing C., Miao Z. (2021). Chalcone: A Privileged Structure in Medicinal Chemistry. Chem. Rev..

[16] Saha C., Naskar R., Chakraborty S. (2017). Antiviral Flavonoids: A Natural Scaffold with Prospects as Phytomedicines against SARS-CoV2. Mini Rev. Med. Chem..

[17] Mezgebe K., Melaku Y., Mulugeta E. (2024). Synthesis and Pharmacological Activities of Chalcone and Its Derivatives Bearing N-Heterocyclic Scaffolds: A Review. ACS Omega.

[18] Elkhalifa D., Al-Hashimi I., Al Moustafa A.-E., Khalil A. (2022). A comprehensive review on the antiviral activities of chalcones. J. Drug Target..

[19] Chen X., Li H., Wang M., Sun D., Lu J., Zhu T., Chen H., Chen L., Liu S. (2021). Discovery of Chalcone Derivatives as Bifunctional Molecules with Anti- SARS-CoV-2 and Anti-inflammatory Activities. J. Nat. Prod..

[20] Herzog A.-M., Göbel K., Marongiu L., Ruetalo N., Alonso M.C., Leischner C., Busch C., Burkard M., Lauer U.M., Geurink P.P. (2024). Compounds derived from *Humulus lupulus* inhibit SARS-CoV-2 papain-like protease and virus replication. Phytomedicine.

[21] Jin Y.-H., Min J.S., Kwon S. (2024). Cardamonin as a p38 MAPK Signaling Pathway Activator Inhibits Human Coronavirus OC43 Infection in Human Lung Cells. Nutrients.

[22] Hu Y., Liu M., Qin H., Lin H., An X., Shi Z., Song L., Yang X., Fan H., Tong Y. (2023). Artemether, Artesunate, Arteannuin B, Echinatin, Licochalcone B and An drographolide Effectively Inhibit SARS-CoV-2 and Related Viruses In Vitro. Front. Cell. Infect. Microbiol..

[23] Shen L., Niu J., Wang C., Huang B., Wang W., Zhu N., Deng Y., Wang H., Ye F., Cen S. (2021). High-Throughput Screening and Identification of Potent Broad-Spectrum Inhibitors of Coronaviruses. J. Virol..

[24] Varinska L., van Wijhe M., Belleri M., Mitola S., Perjesi P., Presta M., Koolwijk P., Ivanova L., Mojzis J. (2019). Anti-angiogenic activity of the flavonoid precursor 4-hydroxychalcone. Eur. J. Pharmacol..

[25] Qu Q., Dai B., Yang B., Li X., Liu Y., Zhang F. (2012). 4-Hydroxychalcone attenuates hyperaldosteronism, inflammation, and ren al injury in cryptochrome-null mice. BioMed Res. Int..

[26] Han X., Zhu Q.-Q., Li Z., He J.-K., Sun Y., Zhong Q.-H., Tang S.-X., Zhang Y.-L. (2014). 4-Hydroxychalcone attenuates AngII-induced cardiac remodeling and dysfunction via regulating PI3K/AKT pathway. Hypertens. Res..

[27] Zhu Q.-C., Wang Y., Liu Y.-P., Zhang R.-Q., Li X., Su W.-H., Long F., Luo X.-D., Peng T. (2011). Inhibition of enterovirus 71 replication by chrysosplenetin and penduletin. Eur. J. Pharm. Sci..

[28] Subramanian A., Narayan R., Corsello S.M., Peck D.D., Natoli T.E., Lu X., Gould J., Davis J.F., Tubelli A.A., Asiedu J.K. (2017). A Next Generation Connectivity Map: L1000 Platform and the First 1,000,000 Profiles. Cell.

[29] Shin H.J., Lee W., Ku K.B., Yoon G.Y., Moon H.W., Kim C., Kim M.H., Yi Y.S., Jun S., Kim B.T. (2024). SARS-CoV-2 aberrantly elevates mitochondrial bioenergetics to induce robust virus propagation. Signal Transduct. Target. Ther..

[30] Chen S., Liu X., Peng C., Tan C., Sun H., Liu H., Zhang Y., Wu P., Cui C., Liu C. (2021). The phytochemical hyperforin triggers thermogenesis in adipose tissue via a Dlat-AMPK signaling axis to curb obesity. Cell Metab..

[31] Ueki I.F., Min-Oo G., Kalinowski A., Ballon-Landa E., Lanier L.L., Nadel J.A., Koff J.L. (2013). Respiratory virus-induced EGFR activation suppresses IRF1-dependent interferon λ and antiviral defense in airway epithelium. J. Exp. Med..

[32] He P., Niu S., Wang S., Shi X., Feng S., Du L., Zhang X., Ma Z., Yu B., Liu H. (2019). Discovery of WS-157 as a highly potent, selective and orally active EG. Acta Pharm. Sin. B.

[33] He P., Jing J., Du L., Zhang X., Ren Y., Yang H., Yu B., Liu H. (2023). Discovery of YS-363 as a highly potent, selective, and orally efficacious EGFR inhibitor. Biomed. Pharmacother..

[34] Jafari R., Almqvist H., Axelsson H., Ignatushchenko M., Lundbäck T., Nordlund P., Martinez Molina D. (2014). The cellular thermal shift assay for evaluating drug target interactions in cells. Nat. Protoc..

[35] Zhang Z., Ye C., Liu J., Xu W., Wu C., Yu Q., Xu X., Zeng X., Jin H., Wu Y. (2021). JaponiconeA induces apoptosis of bortezomib-sensitive and -resistant myeloma cells in vitro and in vivo by targeting IKK. Cancer Biol. Med..

[36] Ma Q., Li R., Pan W., Huang W., Liu B., Xie Y., Wang Z., Li C., Jiang H., Huang J. (2020). Phillyrin (KD-1) exerts anti-viral and anti-inflammatory activities against novel coronavirus (SARS-CoV-2) and human coronavirus 229E (HCoV-229E) by suppressing the nuclear factor kappa B (NF-κB) signaling path way. Phytomedicine.

[37] Xie P., Fang Y., Shen Z., Shao Y., Ma Q., Yang Z., Zhao J., Li H., Li R., Dong S. (2021). Broad antiviral and anti-inflammatory activity of Qingwenjiere mixture against SARS-CoV-2 and other human coronavirus infections. Phytomedicine.

[38] Venkataraman T., Frieman M.B. (2017). The role of epidermal growth factor receptor (EGFR) signaling in SARS coronavirus-induced pulmonary fibrosis. Antivir. Res..

[39] Channappanavar R., Perlman S. (2017). Pathogenic human coronavirus infections: Causes and consequences of cytokine storm and immunopathology. Semin. Immunopathol..

[40] Huang C., Wang Y., Li X., Ren L., Zhao J., Hu Y., Zhang L., Fan G., Xu J., Gu X. (2020). Clinical features of patients infected with 2019 novel coronavirus in Wuhan, China. Lancet.

[41] Williams A.E., Chambers R.C. (2014). The mercurial nature of neutrophils: Still an enigma in ARDS?. Am. J. Physiol. Lung Cell. Mol. Physiol..

