# Supplementary material for: 4-Hydroxychalcone Inhibits Human Coronavirus HCoV-OC43 by Targeting EGFR/AKT/ERK1/2 Signaling Pathway
Source: Viruses. 2025 Jul 23;17(8):1028. doi: 10.3390/v17081028 (PMC12390396; doi:10.3390/v17081028)
Supplement: Supplementary file 1 [file viruses-17-01028-s001.zip › viruses-3744726-supplementary.pdf]

Supplemental materials

Supplementary Table S1. SAR studies of chalcone and its derivatives against human coronavirus HCoV-OC43

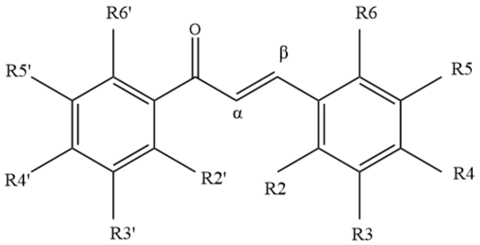

Backbone A
$(\Delta^{\alpha,\beta})$

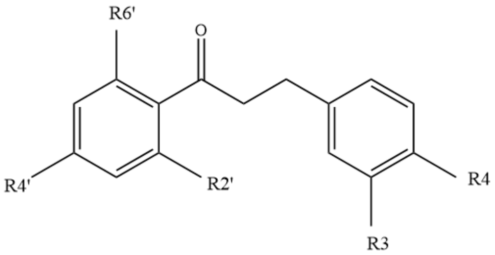

Backbone B

No.	Compound	Structure											IC_{50} (μM) [a]	CC_{50} (μM)[b]
		Backbone	R2	R3	R4	R5	R6	R2'	R3'	R4'	R5'	R6'		
1	Chalcone	A	-H	-H	-H	-H	-H	-H	-H	-H	-H	-H	>10	>50
2	trans-Chalcone	A	-H	-H	-H	-H	-H	-H	-H	-H	-H	-H	4.48	15.02
3	4-Hydroxychalcone	A	-H	-H	-OH	-H	-H	-H	-H	-H	-H	-H	1.83	24.63
4	trans-4-Hydroxychalcone	A	-H	-H	-OH	-H	-H	-H	-H	-H	-H	-H	1.92	32.25
5	Isobavachalcone	A	-H	-H	-OH	-H	-H	-OH	$-C_5H_9$	-OH	-H	-H	>10	>10
6	Licochalcone C	A	$-OCH_3$	$-C_5H_9$	-OH	-H	-H	-H	-H	-OH	-H	-H	>5	28.49
7	2-Hydroxychalcone	A	-OH	-H	-H	-H	-H	-H	-H	-H	-H	-H	NA	—
8	4- Methoxychalcone	A	-H	-H	$-OCH_3$	-H	-H	-H	-H	-H	-H	-H	NA	—
9	4'-Methoxychalcone	A	-H	-H	-H	-H	-H	-H	-H	$-OCH_3$	-H	-H	NA	—

10	4,4'-Dimethoxychalcone	A	-H	-H	$-OCH_3$	-H	-H	-H	-H	$-OCH_3$	-H	-H	NA	—
11	Pinocembrin chalcone	A	-H	-H	-H	-H	-H	-OH	-H	-OH	-H	-OH	NA	—
12	Flavokawain B	A	-H	-H	-H	-H	-H	-OH	-H	OCH_3	-H	$-OCH_3$	NA	—
13	Alpinetin chalcone	A	-H	-H	-H	-H	-H	-OH	-H	-OH	-H	$-OCH_3$	NA	—
14	Echinatin	A	-OCH3	-H	-OH	-H	-H	-H	-H	-OH	-H	-H	NA	—
15	2',3,4,4'-tetrahydroxy Chalcone	A	-H	-OH	-OH	-H	-H	-OH	-H	-OH	-H	-H	NA	—
16	2',4,4',6'-Tetrahydroxychalcone	A	-H	-H	-OH	-H	-H	-OH	-H	-OH	-H	-OH	NA	—
17	Bavachalcone	A	-H	-H	-OH	-H	-H	-OH	-H	-OH	$-C_5H_9$	-H	NA	—
18	Licochalcone A	A	-H	$-C_5H_9$	-OH	-H	$-OCH_3$	-H	-H	-OH	-H	-H	NA	—
19	Licochalcone B	A	$-OCH_3$	-OH	-OH	-H	-H	-H	-H	-OH	-H	-H	NA	—
20	4'-O-Methylbavachalcone	A	-H	-H	-OH	-H	-H	-OH	-H	$-OCH_3$	$-C_5H_9$	-H	NA	—
21	Naringin dihydrochalcone	B	-H	-H	-OH	-H	-H	-OH	-H	$-OC_{12}H_{21}O_9$	-H	-OH	NA	—
22	Neohesperidin Dihydrochalcone	B	-H	-OH	$-OCH_3$	-H	-H	-OH	-H	$-OC_{12}H_{21}O_9$	-H	-OH	NA	—

[a]: IC_{50} was obtained from the *in vitro* antiviral assay against human coronavirus HCoV-OC43. NA: Not activity;
b: CC_{50} was obtained from the cytotoxicity assay with RD cells. —: Not test.
